# Two Japanese families with familial pancreatic cancer with suspected pathogenic variants of *CDKN2A*: a case report

**DOI:** 10.1186/s13053-024-00283-7

**Published:** 2024-07-03

**Authors:** Yoshimi Kiyozumi, Hiroyuki Matsubayashi, Akiko Todaka, Ryo Ashida, Seiichiro Nishimura, Nobuhiro Kado, Satomi Higashigawa, Rina Harada, Eiko Ishihara, Yasue Horiuchi, Goichi Honda, Hirotsugu Kenmotsu, Masakuni Serizawa, Kenichi Urakami

**Affiliations:** 1 Division of Genetic Medicine Promotion, Shizuoka Cancer Center, 1007, Shimonagakubo, Nagaizumi, Suntogun, Shizuoka 411-8777 Japan; 2 Division of Gastrointestinal Oncology, Shizuoka Cancer Center, 1007, Shimonagakubo, Nagaizumi, Suntogun, Shizuoka 411-8777 Japan; 3 Division of Hepato-Biliary-Pancreatic Surgery, Shizuoka Cancer Center, 1007, Shimonagakubo, Nagaizumi, Suntogun, Shizuoka 411-8777 Japan; 4 Research Institute, of Shizuoka Cancer Center, 1007, Shimonagakubo, Nagaizumi, Suntogun, Shizuoka 411-8777 Japan; 5 Department of Medical Oncology and Hematology, Oita University Faculty of Medicine, 1-1, Idaigaoka, Hasama-machi, Yufu, Oita 879-5593 Japan

**Keywords:** Familial atypical multiple mole melanoma syndrome, Familial pancreatic cancer, *CDKN2A*, Germline finding, Genetic counseling

## Abstract

**Background:**

Germline mutations in *CDKN2A* result in Familial Atypical Multiple Mole Melanoma Syndrome (FAMMM) (OMIM #155,601), which is associated with an increased risk of pancreatic ductal adenocarcinoma and melanoma. FAMMM has been reported globally, but it is quite rare in Japan. We report two families with familial pancreatic cancer with suspected pathogenic variants of *CDKN2A* that were incidentally identified through comprehensive genomic profiling.

**Case presentation:**

The first case is a 74-year-old woman with a diagnosis of pancreatic carcinoma with multiple liver metastases. She had family histories of pancreatic cancer, but no personal or family history of malignant melanoma. Whole exon sequencing detected a germline *CDKN2A* variant evaluated as likely pathogenic. The results were disclosed to her daughters after she died, and the same *CDKN2A* variant was detected in one of the daughter. The daughter was referred to a nearby hospital for her clinical management. The second case is a 65-year-old man with pancreatic ductal adenocarcinoma. He had family histories of pancreatic cancer, but no personal or family history of malignant melanoma. He underwent a comprehensive genomic profiling test using pancreatic cancer tissue, and detected a presumed germline pathogenic variant of *CDKN2A*. Germline testing confirmed the same *CDKN2A* variant. Genetic analysis of his relatives produced negative results. Other blood relatives are scheduled for genetic analysis in the future. We report two families with familial pancreatic cancer with suspected pathogenic variants of *CDKN2A* that were incidentally identified through comprehensive genomic profiling.

**Conclusions:**

In current Japanese precision medicine, comprehensive genetic analysis can reveal rare genetic syndromes and offer us the opportunity to provide health management for patients and their relatives. However, gene-specific issues are raised in terms of the evaluation of a variant’s pathogenicity and the extent of surveillance of the at-risk organs due to a lack of genetic and clinical data concerning *CDKN2A* variant carriers in Japan.

## Background

Familial pancreatic cancer (FPC) occurs in families with at least a pair of first-degree relatives with pancreatic ductal adenocarcinoma and accounts for 5–10% of all pancreatic cancer patients [1]. Overall, 10–20% of FPCs are reported to have germline pathogenic variants of several different genes, including *STK11*, *PRSS1*, *CDKN2A*, *BRCA2*, *BRCA1*, *PALB2*, *ATM* and *MMR* (*MLH1*, *MSH2*, *MSH6*, *PMS2*) [2, 3]. In particular, *STK11* and *PRSS1* are associated with an especially high risk of developing pancreatic cancer, but variants in these genes are very rare [1]. Carriers of pathogenic variants of *CDKN2A* account for 2.5% of FPC cases, ranking second to carriers of *BRCA2* variants (3.7%) in the U.S.A [4]. *CDKN2A* encodes the p16Ink4a protein and is a major causative gene for familial atypical multiple mole melanoma syndrome (FAMMM), an autosomal dominant syndrome [5, 6]. The p16Ink4a protein acts at the G1/S checkpoint in the cell cycle, where it inhibits the cyclin-dependent kinases CDK4 and CDK6, thereby preventing tumorigenesis caused by phosphorylation of the RB1 protein [5]. The penetrance of melanoma in families with *CDKN2A* pathogenic variants is estimated to range from 58 to 92% by age 80, and the penetrance of pancreatic cancer is estimated at 17–21% by age 70 [7] to 75 [5] in Western Countries. However, to date, FPCs with germline *CDKN2A* pathogenic variants have only rarely been reported in Japan. We herein report two Japanese FPC cases with suspected pathogenic variants of *CDKN2A*.

## Case presentation

### Case 1

**Fig. 1:**
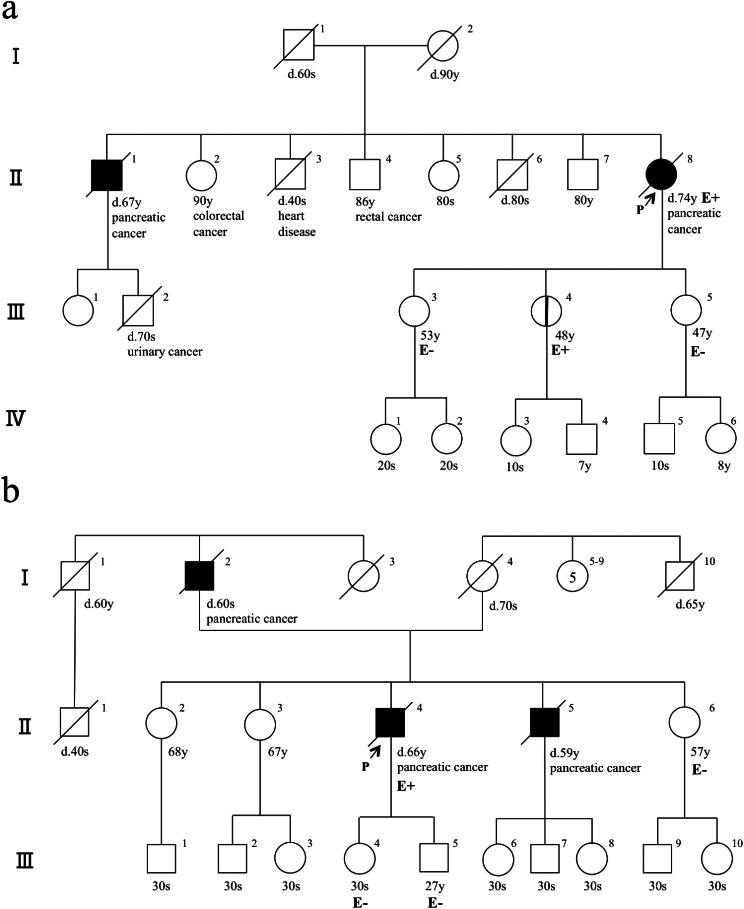
Family trees. (**a**) Case 1 is corresponding to II-8. The patient’s brother died of pancreatic cancer at the age of 67. Malignant melanoma was not recognized in this family. E: *CDKN2A* c.150G > T (p.Gln50His). (**b**) Case 2 is corresponding to II-4. The patient’s father died of pancreatic cancer in his 60s, and his brother died of pancreatic cancer at age 59. The patient had no personal or family history of malignant melanoma. E: *CDKN2A* c.67G > T (p.Gly23Cys). P: proband

A 74-year-old woman (Fig. 1a, II-8) with a diagnosis of pancreatic carcinoma with multiple liver metastases was referred to our hospital. Adenocarcinoma was confirmed by endoscopic ultrasound guided-fine needle biopsy (EUS-FNB) and subsequent histology. Her family history included a brother with pancreatic cancer and two compatriots with colorectal cancer, but she had no personal or family history of malignant melanoma. Microsatellite instability (MSI) analyses using pancreatic cancer tissue demonstrated microsatellite stability (MSS), and immunostaining of mismatch repair (MMR-IHC) proteins revealed proficient expression. She underwent clinical genomic research, and whole exon sequencing detected a germline *CDKN2A* variant evaluated as likely pathogenic based on the criteria of the American College of Medical Genetics and Genomics (ACMG) (Table 1). The patient was administered systemic chemotherapy with gemcitabine and nab-paclitaxel, but she died four months later due to disease progression. Since her death occurred before the genetic analysis results were returned, the results were disclosed to her three daughters (Fig. 1a, III-3, III-4, and III-5) in accordance with her wishes described on the consent form. All three daughters underwent genetic analysis, and the same *CDKN2A* variant was detected in the second daughter (III-4). Since the second daughter lives far from our hospital, she was referred to a nearby hospital for her clinical management. She was offered a multi-institutional prospective surveillance study [8] among kindreds with familial pancreatic cancer and individuals with hereditary pancreatic cancer syndromes at the nearby hospital where she was referred.

**Table 1:** Evaluation of pathogenicity of the germline *CDKN2A* variants detected in the familial pancreatic cancer patients

		Case 1	Case 2
Germline *CDKN2A* variant		c.150G> T (p.Gln50His)	c.67G> T (p.Gly23Cys)
rs No.		rs1057519882	rs1131691186
Location (GRCh37)		chr9: 21,974,677	chr9: 21,974,760
Interpretation of pathogenicity			
ClinVar [review status]		Uncertain significance [★★☆☆]	Conflicting interpretations Likely pathogenic (4); Uncertain significance (1) [★☆☆☆]
HGMD		No registration	Disease causing mutation
ACMG guideline		Likely Pathogenic (PM1, PM2, PM5, PP3)	Likely Pathogenic (PM1, PM2, PM5, PP3)
Registered clinical laboratory		Uncertain significance	Likely pathogenic
Minor allele frequency	jMorp8.3	No registration	No registration
	gnomAD	No registration	No registration
in silico analysis (score)	Polyphen2	Pathogenic Supporting (0.962)	Pathogenic Supporting (0.999)
	SIFT	Pathogenic Supporting (0.089)	Pathogenic Supporting (0)
	M-CAP	Pathogenic Supporting (0.495)	Pathogenic Moderate (0.765)

ClinVar (https://www.ncbi.nlm.nih.gov/clinvar/), HGMD (https://www.hgmd.cf.ac.uk/ac/index.php), database checked in April, 2024

### Case 2

A 65-year-old man (Fig. 1b, II-4) with a complaint of obstructive jaundice was diagnosed with pancreatic ductal adenocarcinoma by a biliary forceps biopsy obtained during his preoperative biliary drainage. He had family histories of pancreatic cancer in his father and brother, but he had no personal or family history of malignant melanoma. Neoadjuvant chemotherapy with gemcitabine and tegafur, gimeracil, oteracil potassium (S-1), and a subsequent pancreaticoduodenectomy were performed, but disease recurrence was observed one year later. During systemic therapy with S-1, he underwent a comprehensive genomic profiling test (FoundationOne^®^ CDx, Foundation Medicine, MA, USA) [9] using surgically resected tissue from his pancreatic cancer (tumor cell ratio: 20%). The genomic profiling test demonstrated somatic pathogenic variants of *CDKN2A* (p.G23C), *CDKN2A* (p.D84Y), *KRAS* (p.G12V), *TET2* (p.S1848*), and *TP53* (p.152 fs*14). According to the Japanese algorithm for secondary finding disclosure in cases of tumor-only analysis (http://sph.med.kyoto-u.ac.jp/gccrc/pdf/k101_kentousiryo_v1.pdf), and with the 46.6% variant allele frequency in mind, the Shizuoka Cancer Center expert panel judged the *CDKN2A* variant (p.G23C) to be a presumed germline pathogenic variant (PGPV). Germline testing performed after genetic counseling confirmed the same *CDKN2A* variant (Table 1). Genetic analysis of his sister (Fig. 1b, II-6), daughter (III-4), and son (III-5) produced negative results. Other blood relatives are scheduled for genetic analysis in the future.

## Discussion

The *CDKN2A* variants detected in the current two cases are located in exon 1a and 2, and may affect the function of p16INK4a [10]. Individuals with pathogenic variants affecting biologically relevant CDKN2A isoforms (i.e., p16INK4A and p14ARF) are recommended for surveillance for both melanoma and pancreatic cancer [11, 12]. For *CDKN2A* variant carriers in Western countries, initiation of full-body skin screening, including the scalp, oral mucosa, genital area, and nails, is recommended at age 10 and followed up every 6–12 months [5]. However, as a markedly lower incidence of malignant melanoma has been reported in Asians than in Caucasians (approximately one tenth) [13, 14], whether Japanese variant carriers require the same level of surveillance is unclear. Recent review paper by Arnold et al. listed the risk factors of melanoma including ethnicity (Caucasian > > Asian, African American), number of atypical nevi, sunbathing habit, UV radiation, and genetics. Markedly lower incidence of melanoma in darker-skinned populations has been explained by the melanocyte’s function to protect skin tissues from DNA damage by ultraviolet (UV) radiation [14, 15].

Individuals with first-degree relatives of FPC and those with several hereditary cancer syndromes who have ≥ 5-fold risk compared with the general population are recommended to undergo surveillance of the pancreas to diagnose and treat earlier stage of pancreatic cancer in Japan [16, 17]. These hereditary cancer syndromes include pathogenic variant carriers of *STK11, PRSS1, BRCA2*, MMR (*MLH1, MSH2, MSH6, PMS2*), *PALB2*, and *ATM.* As the *CDKN2A*/p16 variant carriers (FAMMM) has rarely been reported and the guidelines for FAMMM have not been established in Japan, the Japanese Clinical Guidelines for Pancreatic Cancer (2022) [16] did not state exactly on the method of surveillance. The international cancer of the pancreas screening (CAPS) consortium proposed a strategy for pancreatic surveillance of high-risk individuals to detect pancreatic cancer at the early stage [12]. According to the CAPS consensus, the best imaging modalities for routine follow-up are endoscopic ultrasound (EUS) and magnetic resonance imaging (MRI) or magnetic resonance cholangiopancreatography (MRCP) because of their higher diagnostic abilities without radiation exposure. For *CDKN2A* variant carriers, pancreatic screening is recommended, as a survival benefit is obtained (resectability: 83%, 5-year survival rate: 32%) [7] when the initial examination is started at the age of 40 years [5, 11]. A Dutch cohort study demonstrated a rapid growth of cancers in the pancreases of *CDKN2A/p16* variant carriers, in which a cystic precursor lesion was absent in nearly half of the cases [18]. This lack of a precursor lesion may hinder the early detection of pancreatic cancer in Japan, as pancreatic surveillance of a variant carrier is not covered by Japanese health insurance without associated clinical findings.

The pathogenicity of the *CDKN2A* variants of the two current cases are now rated as likely pathogenic according to the ACMG guideline; however, the variant in case 1 is rated as having uncertain significance and that in case 2 as conflicting interpretation [likely pathogenic (4) and uncertain significance (1)] by the ClinVar (Table 1). The *CDKN2A* variants synthesizing the other amino acids (p.Gly23Arg, p.Gly23Ser), located at the same codon as in the case 2 (p.Gly23Cys), have been reported as pathogenic [10, 19]. Although small in proportion, the evaluation of variants of uncertain significance (VUS) classified by the ClinVar database can change to likely pathogenic/pathogenic according to the time course [20] and additional clinical and/or functional information. The rate of VUS is still higher in Asians and Hispanics than in Caucasians when testing hereditary tumor-related genes [21]. Segregation assays are useful for clarifying the pathogenicity of VUS; however, as in the current cases, genetic testing for patients with past pancreatic cancer is quite difficult due to their poor prognosis [22]. The lifetime risk of pancreatic cancer in *CDKN2A* pathogenic variant carriers is high (17–21% at 70–75 years of age [5, 7]), however with this incidence, segregation assays may result in false negative even in prospectively followed cohorts. Testing methods that can evaluate variant’s pathogenicity, such as MSI/MMR-IHC for Lynch syndrome, are also required for *CDKN2A* variants.

These issues are also reflected in the secondary finding algorithm in Japanese precision cancer medicine. In addition to their risks for melanoma and pancreatic cancer, carriers of *CDKN2A* variants may face moderate risks for breast, lung, and esophageal cancer [5, 23]. No specific physical findings, such as skin fibromas and café-au-lait spots in neurofibromatosis type 1, have been reported in *CDKN2A* variant carriers. Based on the data of MSK-IMPACT (a cancer genome study done at Memorial Sloan Kettering Cancer Center), the European Society for Medical Oncology (ESMO) reported that the germline conversion rate of *CDKN2A* variants was limited, at 4.3% (29/676), and that the characteristic finding of germline *CDKN2A* variant carriers was an early tumor onset (< 30 years of age) [24]. However, their analysis revealed that the patients who developed a tumor at < 30 years old accounted for < 20% of the total *CDKN2A* variant carriers (19.1%, 4/21). Pancreatic cancer, which represents the second largest proportion of cancer types undergoing Japanese precision cancer medicine (https://for-patients.c-cat.ncc.go.jp/registration_status/), develops at a median age of 60 years old (mostly after 50 years old) in *CDKN2A* variant carriers [7]. Hence, if using this recommendation, the majority of the true variant carriers will be missed by tumor-only comprehensive genomic profiling (CGP) in Japan. A prospective study for Dutch *CDKN2A* variant carriers reported that the outcomes of the pancreatic cancer patients were improved by the detection through surveillance (resectability: 83%, 5-year survival rate: 32%) [7]. Recent advance of precision cancer medicine increases the opportunity to detect PGPVs of *CDKN2A/p16* in pancreatic cancer patients. We need to make good use of CGP data and give feedback to the patients and their relatives.

In this case report, we evaluated two families with FPC and suspected pathogenic variants of *CDKN2A* incidentally identified through comprehensive genomic profiling. Gene-specific issues are raised in the evaluation of variant pathogenicity, conditions to select PGPV, extent of surveillance, and the extent of at-risk organs. Accumulation of genetic and clinical data is necessary to obtain the true incidence, penetrance, and phenotype of this rare inherited cancer syndrome.

## Data Availability

No datasets were generated or analysed during the current study.

## Abbreviations

FAMMM: Familial Atypical Multiple Mole Melanoma Syndrome
FPC: Familial pancreatic cancer
MSI: Microsatellite instability
MSS: microsatellite stability
MMR-IHC: immunostaining of mismatch repair
ACMG: The American College of Medical Genetics and Genomics
PGPV: presumed germline pathogenic variant
CAPS: The international cancer of the pancreas screening
EUS: endoscopic ultrasound
MRI: magnetic resonance imaging
MRCP: magnetic resonance cholangiopancreatography
VUS: variants of uncertain significance
ESMO: The European Society for Medical Oncology
CGP: comprehensive genomic profiling

## References

[1] Matsubayashi H, Takaori K, Morizane C, Maguchi H, Mizuma M, Takahashi H (2017). Familial pancreatic cancer: Concept, management and issues. World J Gastroenterol.

[2] Chaffee KG, Oberg AL, McWilliams RR, Majithia N, Allen BA, Kidd J (2018). Prevalence of germ-line mutations in cancer genes among pancreatic cancer patients with a positive family history. Genet Med.

[3] Takai E, Nakamura H, Chiku S, Kubo E, Ohmoto A, Totoki Y (2022). Whole-exome sequencing reveals new potential susceptibility genes for Japanese familial pancreatic Cancer. Ann Surg.

[4] Zhen DB, Rabe KG, Gallinger S, Syngal S, Schwartz AG, Goggins MG (2015). BRCA1, BRCA2, PALB2, and CDKN2A mutations in familial pancreatic cancer: a PACGENE study. Genet Med.

[5] Eckerle Mize D, Bishop M, Resse E, Sluzevich J. Familial Atypical Multiple Mole Melanoma Syndrome. In: Cancer Syndromes. edn. Edited by Riegert-Johnson DL, Boardman LA, Hefferon T, Roberts M. Bethesda (MD): National Center for Biotechnology Information (US) Copyright © 2009, Douglas L Riegert-Johnson.; 2009. 21249757

[6] Santillan AA, Cherpelis BS, Glass LF, Sondak VK (2009). Management of familial melanoma and nonmelanoma skin cancer syndromes. Surg Oncol Clin N Am.

[7] Klatte DCF, Boekestijn B, Wasser M, Feshtali Shahbazi S, Ibrahim IS, Mieog JSD (2022). Pancreatic Cancer surveillance in carriers of a germline CDKN2A pathogenic variant: yield and outcomes of a 20-Year prospective Follow-Up. J Clin Oncol.

[8] Hijioka S, Morizane C, Takaori K, Okusaka T (2022). Study protocol for a multi-institutional prospective surveillance study among kindreds with familial pancreatic cancer and individuals with hereditary pancreatic cancer syndrome: the Diamond Study. Pancreatology.

[9] Ebi H, Bando H. Precision Oncology and the Universal Health Coverage System in Japan. JCO Precis Oncol. 2019;3. 10.1200/PO.19.00291 PMC7446489 32923862

[10] Overbeek KA, Rodríguez-Girondo MD, Wagner A, van der Stoep N, van den Akker PC, Oosterwijk JC (2021). Genotype-phenotype correlations for pancreatic cancer risk in Dutch melanoma families with pathogenic CDKN2A variants. J Med Genet.

[11] Daly MB, Pal T, Maxwell KN, Churpek J, Kohlmann W, AlHilli Z (2023). NCCN Guidelines^®^ insights: Genetic/Familial High-Risk Assessment: breast, ovarian, and pancreatic, Version 2.2024. J Natl Compr Canc Netw.

[12] Goggins M, Overbeek KA, Brand R, Syngal S, Del Chiaro M, Bartsch DK (2020). Management of patients with increased risk for familial pancreatic cancer: updated recommendations from the International Cancer of the pancreas Screening (CAPS) Consortium. Gut.

[13] Matsuda T, Marugame T, Kamo K, Katanoda K, Ajiki W, Sobue T (2012). Cancer incidence and incidence rates in Japan in 2006: based on data from 15 population-based cancer registries in the monitoring of cancer incidence in Japan (MCIJ) project. Jpn J Clin Oncol.

[14] Arnold M, Singh D, Laversanne M, Vignat J, Vaccarella S, Meheus F (2022). Global Burden of Cutaneous Melanoma in 2020 and projections to 2040. JAMA Dermatol.

[15] Hughes BK, Bishop CL. Current understanding of the role of senescent melanocytes in skin ageing. Biomedicines. 2022;10. 10.3390/biomedicines10123111 PMC9775966 36551868

[16] Okusaka T, Nakamura M, Yoshida M, Kitano M, Ito Y, Mizuno N (2023). Clinical practice guidelines for pancreatic Cancer 2022 from the Japan Pancreas Society: a synopsis. Int J Clin Oncol.

[17] Matsubayashi H, Morizane C. Familial and hereditary pancreatic cancer in Japan. Fam Cancer. 2024. 10.1007/s10689-024-00395-y 38733422

[18] Ibrahim IS, Wasser MN, Wu Y, Inderson A, de Cappel VTN, Morreau WH (2018). High growth rate of pancreatic ductal adenocarcinoma in CDKN2A-p16-Leiden Mutation Carriers. Cancer Prev Res (Phila).

[19] Gensini F, Sestini R, Piazzini M, Vignoli M, Chiarugi A, Brandani P (2007). The p.G23S CDKN2A founder mutation in high-risk melanoma families from Central Italy. Melanoma Res.

[20] Shah N, Hou YC, Yu HC, Sainger R, Caskey CT, Venter JC (2018). Telenti A Identification of Misclassified ClinVar variants via Disease Population Prevalence. Am J Hum Genet.

[21] Caswell-Jin JL, Gupta T, Hall E, Petrovchich IM, Mills MA, Kingham KE (2018). Racial/ethnic differences in multiple-gene sequencing results for hereditary cancer risk. Genet Med.

[22] Mizrahi JD, Surana R, Valle JW (2020). Shroff RT pancreatic cancer. Lancet.

[23] Borg A, Sandberg T, Nilsson K, Johannsson O, Klinker M, Måsbäck A (2000). High frequency of multiple melanomas and breast and pancreas carcinomas in CDKN2A mutation-positive melanoma families. J Natl Cancer Inst.

[24] Kuzbari Z, Bandlamudi C, Loveday C, Garrett A, Mehine M, George A (2023). Germline-focused analysis of tumour-detected variants in 49,264 cancer patients: ESMO Precision Medicine Working Group recommendations. Ann Oncol.

